# Three suspected cases of sugammadex-induced anaphylactic shock

**DOI:** 10.1186/1471-2253-14-92

**Published:** 2014-10-17

**Authors:** Tomonori Takazawa, Yukinari Tomita, Nagahide Yoshida, Akihiro Tomioka, Tatsuo Horiuchi, Chie Nagata, Masaki Orihara, Makiko Hardy Yamada, Shigeru Saito

**Affiliations:** Department of Anesthesiology, Gunma University Graduate School of Medicine, 3-39-22 Showa-machi, Maebashi, 371-8511 Japan; Department of Anesthesiology, Isesaki Municipal Hospital, 12-1 Tsunatorihoncho, Isesaki, 372-0817 Japan; Department of Anesthesiology, Saiseikai Maebashi Hospital, 564-1 Kamishinden-machi, Maebashi, 971-0821 Japan; Department of Anesthesiology, Gunma Chuo General Hospital, 1-7-13 Kouuncho, Maebashi, 371-0025 Japan

**Keywords:** Sugammadex, Anaphylactic shock, General anesthesia

## Abstract

**Background:**

Sugammadex has a unique mechanism of action and is widely used because of its safety and efficacy. A few recent reports have described allergic reactions to clinical doses of sugammadex. We hereby describe another series of cases of possible anaphylaxis to sugammadex.

**Case presentation:**

We present three suspected cases of sugammadex-induced anaphylactic shock, including a 13-year-old boy who underwent laparoscopic appendectomy, a 75-year-old woman who underwent left knee arthroplasty, and a 34-year-old man who underwent left pansinectomy for sinobronchitis. All three patients received general anesthesia with rocuronium and their tracheas were intubated. Shortly after injection of sugammadex for reversal of rocuronium, all of them experienced a decrease in blood pressure along with mucocutaneous erythema. In the most severe case, reintubation after extubation was required due to difficulty in manual ventilation. All patients recovered with anti-allergic therapy. On later investigation, all three patients had a positive skin reaction to sugammadex.

**Conclusion:**

Our results suggest that physicians using sugammadex should be aware of the possibility of sugammadex-induced anaphylaxis.

## Background

Sugammadex is widely used in more than 60 countries, including the European Union and Japan, for reversal of the effects of the steroidal neuromuscular blocking agents, rocuronium and vecuronium. Sugammadex acts by encapsulation of the rocuronium molecule, such that no more rocuronium is available for binding with nicotinic acetylcholine receptors in the neuromuscular junction. This action of sugammadex for antagonizing rocuronium is completely different from that of acetylcholinesterase inhibitors, such as neostigmine. Sugammadex is generally thought to be a relatively safe and well-tolerated drug, partly due to the lack of some undesirable effects [1, 2]. However, confirmed cases of allergic reactions to clinical doses of sugammadex were recently reported [3–8]. We describe 3 cases in which allergic and positive skin test reactions to sugammadex were observed. They include a life-threatening case in which reintubation after extubation was required, probably due to sugammadex-induced anaphylaxis.

## Case presentation

### Case 1

A 13-year old, 40 kg, 159 cm boy underwent laparoscopic appendectomy. Anesthesia was induced with 80 mg propofol, 0.33 μg/kg/min remifentanil, and 30 mg rocuronium. After an uneventful intubation, anesthesia was maintained with sevoflurane and remifentanil. About 15 minutes after intubation, 50 mg flurbiprofen axetil was administered. During the surgery, oxygen saturation was maintained in the high 90% range and ETCO_2_ was within normal limits. Baseline blood pressure (BP) was approximately 116/78 mm Hg, remaining in the 90/40 mm Hg range for most of the case. Heart rate (HR) remained at 50 to 60 bpm. The total operation time was 118 minutes. Before extubation, sugammadex 2 mg/kg was injected to antagonize residual neuromuscular blockade. Shortly after the injection, the patient’s trachea was extubated when he was able to breathe spontaneously. After extubation, the patient demonstrated global swelling of the face and a low consciousness level. At this point, his blood pressure fell to unmeasurable values and he developed tachycardia of between 150 and 160 bpm. Suspecting a possible allergic reaction to sugammadex, he was treated with 100 mg hydrocortisone and 0.1 mg adrenaline IV. The patient needed to be re-intubated because of difficulty with bag mask ventilation. Blood gas analysis showed hypercapnea (PCO_2_ = 66.4 mmHg). His trachea was re-intubated after re-induction of anesthesia with 80 mg propofol and 50 mg rocuronium. Additional treatment with 200 mg aminophylline and 200 mg hydrocortisone was given after the intubation, because the occurrence of an asthmatic attack could not be excluded. The patient stayed in the ICU until his trachea was extubated 17 hours after the event. A prick test with dilutions of propofol, rocuronium, ropivacaine, flurbiprofen axetil, and sugammadex was performed 7 weeks after the operation, which showed an immediate reaction only to sugammadex (Table 1).

**Table 1:** **Results of postoperative allergy testing in the three patients with suspected anaphylaxis**

	Type of allergy test	Drug	Concentration 1:1000	Concentration 1:100	Wheal (mm)	Flare (mm)
Case 1	Prick test	Saline	-	N/A	0	0
		Propofol	-		0	0
		Rocuronium	-		0	0
		Ropivacaine	-		0	0
		Flurbiprofen axetil	-		0	0
		Sugammadex	+		12	25
Case 2	Intradermal test	Saline	-	-	0	0
		Propofol	-	-	0	0
		Rocuronium	-	-	0	0
		Flurbiprofen axetil	-	-	0	0
		Sugammadex	-	+	8	25
Case 3	Intradermal test	Saline	-	-	0	0
		Flurbiprofen axetil	-	-	0	0
		Pentazocine	-	-	0	0
		Sugammadex	+/-	+	10	35

Vial concentrations: propofol 10 mg/ml, rocuronium 10 mg/ml, ropivacaine 7.5 mg/ml, flurbiprofen axetil 10 mg/ml, pentazocine 15 mg/ml, sugammadex 100 mg/ml.

### Case 2

A 75-year-old, 71 kg, 152 cm woman underwent left knee arthroplasty. General anesthesia was induced with 130 mg propofol, 0.2 μg/kg/min remifentanil, and 40 mg rocuronium. Following tracheal intubation, anesthesia was maintained with sevoflurane and remifentanil. Ten minutes after induction of general anesthesia, her BP and HR decreased to 70/50 mmHg and 50 bpm, respectively. Incremental doses of ephedrine (8 mg × 2), 0.2 mg phenylephrine and 0.5 mg atropine were administered intravenously. Both BP and HR responded well to these treatments and increased to 150/70 mm Hg and 75 bpm, respectively. Twenty minutes before the end of surgery, 50 mg flurbiprofen axetil was administered. The total operation time was 99 minutes. Before extubation, sugammadex 2.8 mg/kg was administered. Three minutes after the injection of sugammadex, her systolic BP fell to 40/undetectable mm Hg, together with thoracic erythema, with no response to 0.2 mg phenylephrine injection. No respiratory symptoms, such as wheezing and high airway resistance, were observed. Suspecting a possible allergic reaction to sugammadex, we intravenously administered 0.5 mg adrenaline, 5 mg chlorpheniramine maleate, 50 mg ranitidine hydrochloride, and 300 mg hydrocortisone. Her BP gradually increased to 160/80 mmHg after these treatments. The patient was transferred to the intensive care unit (ICU) without extubation. Her trachea was extubated 15 hours later in the ICU when she was fully awake and hemodynamically stable. She was transferred out of the ICU the following morning. Four weeks after the operation, an intradermal allergy test with dilutions of propofol, rocuronium, flurbiprofen axetil, and sugammadex was performed. A 1:1000 dilution of all the drugs showed a negative reaction. However, a 1:100 dilution of sugammadex resulted in a positive reaction (Table 1).

### Case 3

A 34-year old, 62 kg, 159 cm man underwent left pansinectomy for sinobronchitis. Anesthesia was induced with 100 mg propofol, 0.25 μg/kg/min remifentanil, and 40 mg rocuronium. Following tracheal intubation, anesthesia was maintained with sevoflurane and remifentanil. During induction and maintenance of anesthesia, BP, oxygen saturation, and ETCO_2_ were within normal ranges. The total operation time was 59 minutes. At the end of surgery, 50 mg flurbiprofen axetil, 15 mg pentazocine, and sugammadex 3.2 mg/kg were injected. One minute after the injection but before extubation, the patient developed intense erythema all over the body, especially on the face and chest. Simultaneously, BP decreased from 100/60 to 70/40 mmHg, with no respiratory symptoms. BP recovered to 100/60 mmHg after 4 mg ephedrine hydrochloride injection, and the patient was transferred to the recovery unit. Erythema disappeared after treatment with 5 mg chlorpheniramine maleate and 50 mg ranitidine hydrochloride administered intravenously. The patient was transferred out of the recovery room 1 hour after the event and discharged home 6 days later. An intradermal test for flurbiprofen axetil, pentazocine, and sugammadex allergy was performed about 7 weeks after the operation. As shown in Table 1, a 1:1000 dilution of sugammadex induced flare, while a 1:100 dilution of sugammadex induced both flare and wheal.

## Discussion

The etiological diagnosis of perioperative anaphylaxis relies on a triad of evidences, including clinical, biological, and allergological evidence [9]. We report here three cases, in which both clinical symptoms and positive skin test reactions were observed in response to sugammadex injection.

The first line of evidence for diagnosing anaphylaxis includes clinical signs. The National Institute of Allergy and Infectious Disease (NIAID) and the Food Allergy and Anaphylaxis Network (FAAN) proposed clinical criteria for diagnosing anaphylaxis [10]. All three of our cases fulfilled these criteria, as evidenced by the acute onset of symptoms, with involvement of the skin and hypotension.

The second line of evidence for diagnosing anaphylaxis is biological assessment, including plasma histamine and tryptase measurements. Typically, anaphylaxis results from mast cell activation, which causes release of mast cell tryptase into the circulation, although a variety of other pathways, including basophil or complement activation, may combine to produce anaphylaxis. We were unable to perform any blood tests in our patients. The currently available tryptase assay has a relatively low sensitivity; hence, some cases of anaphylaxis might be missed [11]. However, given the high specificity of tryptase assay [12], such assessment should be done for diagnosing anaphylaxis. During anaphylaxis, tryptase peaks by approximately 1 h and its half-life in the circulation is about 2 h [11], suggesting that elevation of tryptase can still be seen at least 1-2 hours after onset of the reaction, by which time the patient should be stabilized.

Skin tests, the third line of evidence, remain the gold standard for the detection of IgE-mediated reactions, and involve exposure of the mast cells of the skin of patients who experience anaphylaxis to the suspected allergen [13]. In previous reports, either skin-prick [3, 4] or intradermal [7, 8] tests were performed to detect hypersensitivity to sugammadex. However, there are no established guidelines with respect to skin testing for hypersensitivity to sugammadex. Moreover, it is still unknown which of the two tests is superior. In our three cases, we performed prick or intradermal tests in the operation room several weeks after the incidents, with drugs to treat anaphylaxis readily available. The results showed a positive reaction to only sugammadex in all cases. We chose relatively low concentrations (i.e., 1:1000 or 1:100) of sugammadex for both prick and intradermal tests to avoid false-positive reactions, because it is known that intradermal tests sometimes produce false-positive reactions [14]. A recent study showed that dilutions of 100 mg/ml sugammadex at 1:77 and 1:770 for intradermal tests did not cause skin irritation or false positive reactions in 11 volunteers, suggesting that 1:100 and higher dilutions of sugammadex are not likely to produce false-positive reactions [15]. However, it was difficult to exclude the possibility that the positive reaction to sugammadex might have been a non-specific cutaneous irritant effect, because of the lack of a positive control skin test with histamine. In addition to skin tests, tests for serum-specific IgE antibodies and basophil activation using flow-cytometry may also be useful for the diagnosis of anaphylaxis [11, 16, 17]. Taken together, the immediate development of anaphylaxis in response to sugammadex and the results of skin tests were highly suggestive of sugammadex-induced anaphylactic shock, although biological assessments and positive control skin tests were lacking.

None of our patients had prior exposure to sugammadex. Cyclodextrins, one of the components of sugammadex preparations, are present in various foods, which may partly explain the cross-reaction with sugammadex. A recent report suggested that the appropriate dose of sugammadex for reversal of rocuronium should be determined by the results of neuromuscular monitoring [18]. However, we did not monitor neuromuscular transmission in our patients.

Sugammadex-induced anaphylaxis typically presents when the patient is already extubated and is being transferred to the hospital bed, PACU, ICU, etc. [3], time points when the patient is typically less monitored. Therefore, rapid diagnosis and appropriate treatment of anaphylaxis, including administration of adrenaline, oxygen, and large volumes of crystalloids, are required. In the current report, patients 1 and 2 were treated with 0.1 and 0.5 mg adrenaline IV, respectively. Although the maximum intravenous bolus dose of adrenaline recommended by recent guidelines for anaphylaxis during anesthesia is variable [17, 19], the IV bolus dose of 0.5 mg adrenaline administered to case 2 appears to be a larger dose than that recommended by guidelines.

The Japanese Society of Anesthesiologists has issued a warning about sugammadex-induced anaphylactic shock three times since March 2011. The latest one, issued in June 2013, included 95 cases of sugammadex-related allergies that occurred between April 2010 and January 2013, although with no incidents of death. In all 95 cases, the relationship between the reaction and sugammadex was definitively ascertained by the anesthesiologists. Seventy-eight out of 95 cases fulfilled the validated criteria for anaphylaxis [10]. The incidence rate of anaphylactic reactions due to sugammadex was estimated as 29 per million cases, based on the estimated number of patients (3.09 millions) in whom sugammadex was injected during the survey period. The alert also pointed out that the incidence rate may have been underestimated, because the survey was based on spontaneous reports from anesthesiologists and not on prospective studies. It is uncertain whether this incidence rate is higher in Japan than in other countries, because there is no epidemiological survey regarding this so far. The other possibility is that these warnings may simply reflect a high level of sugammadex usage in Japan. The drug company, MSD (Tokyo, Japan), has reported that sugammadex usage in Japan in 2010, in terms of monetary value, was more than 4 times higher than that in Spain, the country that showed the second highest usage in the world.

## Conclusion

We would advise physicians using sugammadex to be aware of the possibility of sugammadex-induced anaphylaxis.

### Consent

Written informed consent was obtained from the patients for publication of this case report. A copy of the written consent is available for review by the Editor of this journal. All treatment and clinical testing were performed in accordance with the ethical standards laid down in the 1964 Declaration of Helsinki and its later amendments.
